# Cocaine Paired Environment Increases SATB2 Levels in the Rat Paraventricular Thalamus

**DOI:** 10.3389/fnbeh.2018.00224

**Published:** 2018-10-02

**Authors:** Ahmad Salti, Galina Apostolova, Kai K. Kummer, Cristina Lemos, Georg Dechant, Rana El Rawas

**Affiliations:** ^1^Experimental Psychiatry Unit, Medical University of Innsbruck, Innsbruck, Austria; ^2^Institute of Molecular Biology, University of Innsbruck, Innsbruck, Austria; ^3^Institute for Neuroscience, Medical University of Innsbruck, Innsbruck, Austria; ^4^Division of Physiology, Medical University of Innsbruck, Innsbruck, Austria

**Keywords:** cocaine, conditioned place preference, SATB2, social interaction, reward, paraventricular thalamus

## Abstract

SATB2 is a DNA binding protein that specifically binds the nuclear matrix attachment region and functions as a regulator of the transcription of large chromatin domains. Unlike its well addressed role during brain development, the role of SATB2 in adult brain is under-investigated. It has been shown that deletion of SATB2 from the forebrain of adult mice significantly impaired long-term memory for contextual fear and object recognition memory. The aim of the present study was to investigate the effects of appetitive stimuli such as cocaine and social interaction (SI) on SATB2 expression in the adult rat brain. For that, we performed conditioned place preference (CPP) to cocaine (15 mg/kg) and to SI, then assessed SATB2 expression in the brain 1 h (24 h after the last conditioning) and 24 h (48 h after the last conditioning) after the CPP test. We found that SATB2 expression in the paraventricular thalamus of rats was increased 1 h after the cocaine CPP test. This increase was selective for the cocaine-paired environment since the SI-paired environment did not increase SATB2 expression in the paraventricular thalamus. Also, the cocaine paired environment-induced increase of SATB2 levels in the paraventricular thalamus was due to cocaine conditioning as the unpaired cocaine group did not show an increase of SATB2 in the paraventricular thalamus. These results suggest that SATB2 in the paraventricular thalamus appears to be involved in the association between cocaine effects and environmental context. Further studies are needed to address the functional role of SATB2 in cocaine conditioning.

## Introduction

Reward-related memories play a crucial role in drug dependence (Hyman et al., 2006). Conditioned place preference (CPP) is one of the experimental protocols that provides information about the rewarding effects of the contextual cues associated with a stimulus (Bardo and Bevins, 2000). In CPP, the subject is paired with drug reward in a specific context during several sessions. Later, when the subject has free choice in a drug-free state between the drug or the non-drug paired context, it spends more time in the drug-paired context (Sanchis-Segura and Spanagel, 2006) and encodes long-term memory of drug reward (Itzhak et al., 2014). Indeed, subjects still prefer the cocaine associated context long after pairing sessions occurred (Fritz et al., 2011). Social interaction (SI) is a natural reward shown to induce place preference (Kummer et al., 2011; El Rawas and Saria, 2015) and to be a beneficial alternative to cocaine (Fritz et al., 2011). SI reward was also reported to have the same reward value as cocaine in rats (Fritz et al., 2011) and to activate almost the same brain regions of the reward circuitry (El Rawas et al., 2012).

SATB2 is a transcription factor of a novel type that binds to the matrix attachment DNA region and functions as a regulator of the transcription of large chromatin domains (Britanova et al., 2005). Unlike its well identified role during brain development (Szemes et al., 2006; Sasaki et al., 2008; Tashiro et al., 2011), the role of SATB2 in adult brain is under-investigated. It has been shown that SATB2 is required for long-term memory formation (Jaitner et al., 2016). The same group generated a novel conditional SATB2 mutant line that lacked SATB2 protein in the cortex and the hippocampus at adult but not young age (Satb2 cKO), and showed that these mice exhibited a significant decrease in freezing during a contextual fear conditioning test when compared to control littermates 24 h after the last fear conditioning training. Furthermore, re-introduction of SATB2 into the hippocampus of Satb2 cKO mice via viral vector injection increased freezing up to control levels, 24 h after the last fear conditioning training. These mice also demonstrated significant deficits in object location memory test and novel object recognition memory tasks when the tests were performed 24 h after the last training. Moreover, late long-term potentiation (LTP) was significantly attenuated in Satb2 cKO mice. Altogether, these findings show that SATB2 has a role in long-term memory processes (Jaitner et al., 2016).

The aim of the present study was to investigate the rewarding effects of cocaine (drug) and SI (natural reward) on SATB2 expression in the adult brain. We proposed that appetitive stimuli such as cocaine and/or SI would alter SATB2 expression. In order to know where SATB2 is expressed in the adult rat brain, we first performed a brain mapping of SATB2 expression and subsequently investigated the effects of the cocaine and SI-paired environment on SATB2 expression in these regions.

## Materials and Methods

### Animals

Male Sprague Dawley (SD) rats (6–8 weeks old; 150–200 g; Charles River, Germany) were housed at controlled environmental conditions (12 h light/dark schedule, at a room temperature of 23 ± 1°C) with food and water supplied* ad libitum*. Experiments were performed during the light phase of the circadian cycle. All experiments were approved by the Austrian National Animal Experiment Ethics Committee (BMWF-66.011/0095-ll/3b/2013).

### Conditioned Place Preference

**Figure 1:**
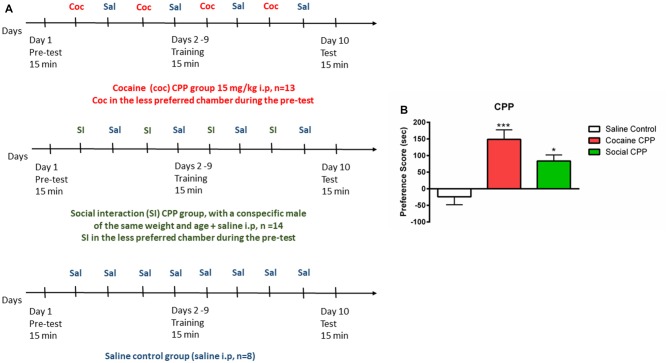
Conditioned place preference (CPP). **(A)** Protocol of acquisition of CPP to cocaine (coc, upper panel), to social interaction (SI, middle panel) and saline control group (lower panel). **(B)** Cocaine or SI produced significant CPP. Preference score (PF) is the difference in time spent in the compartment associated with cocaine or SI on the test day (or the less preferred compartment for the saline control group) minus the time spent in the same stimulus-associated compartment (or the less preferred compartment for the saline control group) on the pre-test day, ***p < 0.001 and *p < 0.05 compared to saline control.

The CPP apparatus consisted of a three-compartment chamber with a middle (neutral) compartment connected to the two outer/conditioning compartments that differed in floor and wall conditions (Salti et al., 2015). The CPP experiment consists in three phases: (1) Pretest, (2) conditioning/training, and (3) test, as described in Figure 1A. Each phase was digitally recorded with a video camera and the time spent in each compartment during the pretest and test was measured offline, by an experimenter blind to the conditions.

Preference was defined by the preference score (PF). PF (SI, cocaine) = Time _TEST_ (stimulus chamber) − Time _PRETEST_ (stimulus chamber); PF (saline) = Time_TEST_ (less preferred chamber during pretest) − Time _PRETEST_ (less preferred chamber during pretest).

As SATB2 was shown to have a role in long-term memory (Jaitner et al., 2016), in the present study the rats from the different treatments were randomly assigned to two time points:

(1) 24 h after the last conditioning, i.e., the animals were sacrificed 1 h after CPP test (CPP cocaine, *n* = 7; CPP SI, *n* = 8; control saline for CPP 1 h, *n* = 4)

(2) 48 h after the last conditioning, i.e., the animals were sacrificed 24 h after the CPP test (CPP cocaine, *n* = 6; CPP SI, *n* = 6; control saline for CPP 24 h, *n* = 4)

To dissociate the effects of pharmacological cocaine injection from the effects of cocaine conditioning in CPP, rats were given the same cocaine treatment of the CPP procedure in the home cage without exposing them to the conditioning chambers. These rats were then sacrificed at the same time point as those that underwent CPP i.e., the unpaired cocaine group that served as a control for the CPP 1 h (*n* = 5), received a total of four cocaine injections and four saline injections (alternate-day-design, one injection per day) in the home cage then was sacrificed 24 h after the last injection. The unpaired saline control group of rats (*n* = 5), received a total of eight saline injections in the home cage (one injection per day) then they were sacrificed 24 h after the last injection.

### Immunohistochemistry

Rats were deeply anesthetized with isoflurane and transcardially perfused. Immunohistochemistry in brain slices was also performed as described in Salti et al. (2015). Briefly, the sections were permeabilized, blocked and then incubated with a rabbit monoclonal SATB2 antibody (1:200, abcam #ab 29446). The sections were subsequently incubated with the secondary biotinylated anti-rabbit antibody (1:200, Vector Laboratories #BA-1000), then with avidin-biotinylated horseradish peroxidase complex (ABC Elite kit) and finally in in 3,3-diaminobenzidine tetrahydrochloride (DAB tablets, Sigma). Afterward the sections were mounted onto Leica Extra adhesive micro slides and allowed to dry before coverslipping. For each brain region, three sections per animal were scanned, using a Zeiss optical microscope set at ×20 magnification equipped with a camera (Axioplan 2 Imaging) interfaced to a PC. Intensity of DAB staining as described by Nguyen et al. (2013) was evaluated by an experimenter blind to treatment conditions using Fiji imaging software. The mean of the intensity value of the three sections/region/animal were included in the statistical analysis.

### Statistical Analyses

All data were expressed as mean ± standard error of the mean (SEM). Differences in PFs and in the intensity of SATB2 staining between the groups (cocaine CPP, SI CPP and saline control rats; unpaired saline and unpaired cocaine rats) were performed by one-way or two-way analysis of variance (ANOVA). Results showing significant overall changes were subjected to Tukey’s Multiple Comparison *post hoc* test. *P* < 0.05 was considered statistically significant.

## Results

### Conditioned Place Preference

In rats, cocaine conditioning and SI conditioning produced significant CPP as compared to the saline control group (Figure 1B, one-way ANOVA, effect: treatment (saline control or cocaine CPP or social CPP), *F*_(2,30)_ = 10.13, *p* = 0.0004). *Post hoc* analysis showed that conditioning with cocaine (cocaine CPP vs. saline control, *p* < 0.001, Cohen’s *d* = 2) or SI (SI CPP vs. saline control, *p* < 0.05, Cohen’s *d* = 1.62) yielded a significantly higher PF as compared to saline control. Finally, the PF for cocaine CPP did not significantly differ from SI CPP (*p* > 0.05) suggesting that SI has the same reward value as cocaine at the dose of 15 mg/kg in rats (Kummer et al., 2014; El Rawas and Saria, 2015).

### SATB2 Expression

In rats, SATB2 was found to be expressed in the prefrontal cortex subregions (Cingulate cortex area- Cg1, Infralimbic- IL and Prelimbic cortex- PrL), field CA1 of the hippocampus, paraventricular thalamic nucleus (PV), arcuate hypothalamic nucleus (Arc), ventromedial hypothalamus (VM), basomedial amygdaloid nucleus (BMA) and posterolateral cortical amygdaloid nucleus (Plco) of rats (Supplementary Figure S1).

### Effects of CPP on SATB2 Expression

**Figure 2:**
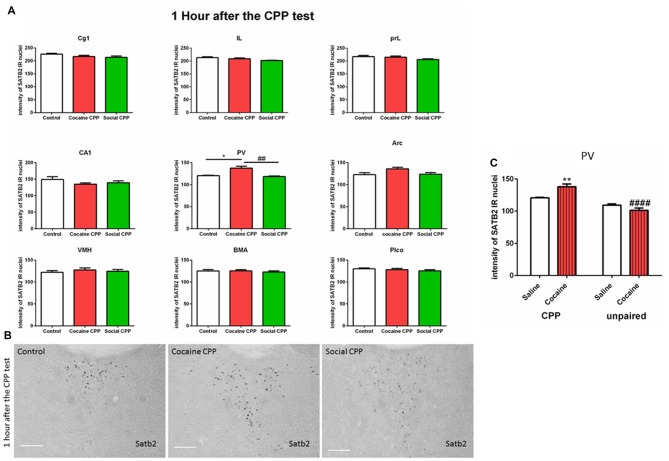
**(A)** SATB2 expression 1 h after the CPP test (24 h after the last conditioning). Cocaine CPP increased SATB2 expression in the paraventricular thalamus. Cg1, cingulate cortex area; IL, infralimbic; PrL, prelimbic cortex; CA1, field CA1 of the hippocampus; PV, paraventricular thalamic nucleus; Arc, arcuate hypothalamic nucleus; VM, ventromedial hypothalamus; BMA, basomedial amygdaloid nucleus and Plco; posterolateral cortical amygdaloid nucleus, *p < 0.05 compared to control; ^##^*p* < 0.01 compared to social CPP. **(B)** Example of immunohistochemistry in the paraventricular thalamus of control, cocaine CPP and social CPP rats 1 h after the CPP test. Scale Bar: 100 μm. **(C)** SATB2 expression in the PV after cocaine CPP and unpaired cocaine. Cocaine CPP but not unpaired cocaine increased SATB2 in the paraventricular thalamus, **p < 0.01 compared to saline control and ^####^*p* < 0.0001 compared to cocaine CPP.

One hour after the CPP test, cocaine CPP increased SATB2 levels in the paraventricular thalamus as compared to saline control and SI CPP associated levels (two-way ANOVA; effect: region, *F*_(8,137)_ = 320.7, *p* < 0.0001; effect: treatment (saline control or cocaine CPP or social CPP), *F*_(2,137)_ = 7.259, *p* = 0.001; interaction (region × treatment), *F*_(16,137)_ = 1.649, *p* = 0.0641). Tukey’s multiple comparisons test showed a significant difference only in the paraventricular thalamus (Figures 2A,B, cocaine CPP vs. saline control, *p* < 0.05, Cohen’s *d* = 2.51; cocaine vs. social CPP, *p* < 0.01, Cohen’s *d* = 2.7; saline control vs. social CPP, *p* > 0.05).

Twenty-four hours after the CPP test, SATB2 expression remained unaltered after cocaine CPP or SI CPP (Supplementary Figure S2; two-way ANOVA; effect: region, *F*_(8,116)_ = 311.2, *p* < 0.0001; effect: treatment (saline control or cocaine CPP or social CPP), *F*_(2,116)_ = 0.4955, *p* = 0.6106; interaction (region × treatment), *F*_(16,116)_ = 0.6545, *p* = 0.8324).

### Cocaine-Paired Environment Increased SATB2 in the Paraventricular Thalamus

In order to investigate whether the increase of SATB2 in the paraventricular thalamus is due to the pharmacological effects of cocaine or due to cocaine conditioning, we compared SATB2 levels obtained in the paraventricular thalamus 1 h after the CPP test with SATB2 levels in the paraventricular thalamus after repeated cocaine treatment in the home cage.

As opposed to cocaine CPP that induced SATB2 levels in the paraventricular thalamus, repeated cocaine injections in the home cage failed to increase SATB2 in the PV as compared to repeated saline injected rats (two-way ANOVA; effect: treatment (CPP or unpaired), *F*_(1,15)_ = 57.29, *p* < 0.0001; effect: drug (saline or cocaine), *F*_(1,15)_ = 2.235, *p* = 0.1557; interaction (treatment × drug), *F*_(1,15)_ = 15.60, *p* = 0.0013). Tukey’s multiple comparisons test showed a significant difference in SATB2 levels after CPP to cocaine as compared to the unpaired cocaine group (Figure 2C, CPP cocaine vs. unpaired cocaine group, *p* < 0.0001, Cohen’s *d* = 4.1; cocaine CPP vs. saline control, *p* < 0.01, Cohen’s *d* = 2.51).

## Discussion

In this study, we showed that the environment paired with cocaine increased SATB2 levels in the paraventricular thalamus and that this increase was selective for the group conditioned with cocaine, as SI CPP did not induce an increase in SATB2 levels in the paraventricular thalamus.

Many studies support the implication of the paraventricular thalamus in drug seeking behavior (Martin-Fardon and Boutrel, 2012; James and Dayas, 2013). Indeed, the lesion of the paraventricular thalamus has been shown to prevent context-induced reinstatement of beer-seeking (Hamlin et al., 2009). Intra-paraventricular thalamus infusion of either tetrodotoxin or cocaine- and amphetamine-regulated transcript resulted in a significant attenuation of drug-primed reinstatement of cocaine seeking behavior (James et al., 2010). Furthermore, transient inactivation of the paraventricular thalamus prevented cue-induced reinstatement of cocaine seeking but not sweetened condensed milk seeking (Matzeu et al., 2015b). Additionally, the activation of PV was positively correlated with cocaine seeking behavior but not with sweetened condensed milk seeking behavior (Matzeu et al., 2015a). Likewise, we found an increase of SATB2 in the paraventricular thalamus after cocaine CPP but not after SI CPP (non-drug), suggesting that the paraventricular thalamus is selectively recruited during cocaine seeking behavior. Browning et al. (2014) have shown that inactivation of the paraventricular thalamus prevented the expression of CPP to cocaine. We propose that SATB2 in the paraventricular thalamus is necessary for the expression of cocaine CPP. Nevertheless, further studies are needed to prove the functional implication of SATB2 in the paraventricular thalamus in cocaine CPP.

In conclusion, we could show that an environment paired with cocaine induced an increase of SATB2 levels in the paraventricular thalamus, a region receiving growing attention in drug seeking behavior. It appears that SATB2 is involved in the association of cocaine effects and the environmental context. Further studies are needed to address the functional role of SATB2 in drugs’ rewarding effects.

## Author Contributions

AS, GA and RER designed the experiments. AS and KK performed the experiments. RER analyzed the data. RER and CL wrote the manuscript. GD provided critical revision of the manuscript.

## Conflict of Interest Statement

The authors declare that the research was conducted in the absence of any commercial or financial relationships that could be construed as a potential conflict of interest.

## Supplementary Material

FIGURE S1 Immunohistochemistry of SATB2 showing the regions in the brain in which SATB2 was expressed. BMA, basomedial amygdaloid nucleus; Arc, arcuate hypothalamic nucleus; VM, ventromedial hypothalamus; PV, paraventricular thalamic nucleus; CA1, field of the hippocampus; Plco, postrolateral cortical amygdaloid nucleus; IL, infralimbic cortex; Cg1, cingulate cortex area 1; PrL, prelimbic cortex. Scale Bar: 100 μm.

FIGURE S2 SATB2 expression 24 h after the CPP test (48 h after the last conditioning). SATB2 expression remained unaltered 24 h after the CPP test to cocaine or social interaction, IL, infralimbic; PrL, prelimbic cortex; CA1, field CA1 of the hippocampus; PV, paraventricular thalamic nucleus; Arc, arcuate hypothalamic nucleus; VM, ventromedial hypothalamus; BMA, basomedial amygdaloid nucleus; and Plco, posterolateral cortical amygdaloid nucleus.

## Abbreviations

Arc: Arcuate hypothalamic nucleus
BMA: Basomedial amygdaloid nucleus
Cg1: Cingulate cortex area 1
CPP: Conditioned place preference
GABA: Gamma aminobutyric acid
I.p: Intraperitoneal
IL: Infralimbic cortex
LTP: Long-term potentiation
PF: Preference score
Plco: Posterolateral cortical amygdaloid nucleus
PrL: Prelimbic cortex
PV: paraventricular thalamus
Satb2 cKO: SATB2 conditional knock out line
SD: Sprague Dawley
SI: Social interaction
VM: Ventromedial hypothalamus.
