# Supplementary figures and images for: Cocaine Paired Environment Increases SATB2 Levels in the Rat Paraventricular Thalamus
Source: Front Behav Neurosci. 2018 Oct 2;12:224. doi: 10.3389/fnbeh.2018.00224 (PMC6190852; doi:10.3389/fnbeh.2018.00224)

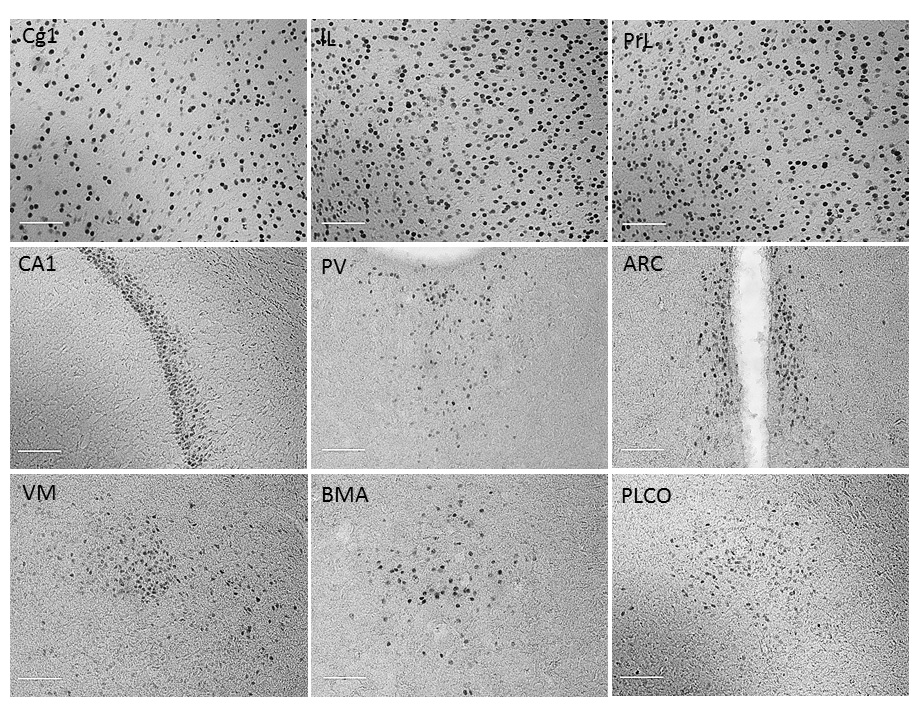

Supplement: FIGURE S1 — Immunohistochemistry of SATB2 showing the regions in the brain in which SATB2 was expressed. BMA, basomedial amygdaloid nucleus; Arc, arcuate hypothalamic nucleus; VM, ventromedial hypothalamus; PV, paraventricular thalamic nucleus; CA1, field of the hippocampus; Plco, postrolateral cortical amygdaloid nucleus; IL, infralimbic cortex; Cg1, cingulate cortex area 1; PrL, prelimbic cortex. Scale Bar: 100 μm. [file Image_1.JPEG]
